# RNA-seq analysis and reconstruction of gene networks involved in response to salinity stress in quinoa (*cv*. Titicaca)

**DOI:** 10.1038/s41598-023-34534-9

**Published:** 2023-05-05

**Authors:** Sahar Sadat Hosseini, Seyedeh Sanaz Ramezanpour, Hassan Soltanloo, Seyed Ebrahim Seifati

**Affiliations:** 1 Department of Plant Breeding and Plant Biotechnology, Gorgan University of Agricultural Sciences and Natural Resources, Gorgan, Golestan Iran; 2 Department of Arid Land and Desert Management, School of Natural Resources and Desert Studies, Yazd University, Yazd, Iran

**Keywords:** Plant genetics, Plant stress responses, Transcriptomics

## Abstract

To better understand the mechanisms involved in salinity stress, the adaptability of quinoa cv. Titicaca—a halophytic plant—was investigated at the transcriptome level under saline and non-saline conditions. RNA-sequencing analysis of leaf tissue at the four-leaf stage by Illumina paired—end method was used to compare salt stress treatment (four days after stress at 13.8 dsm^−1^) and control. Among the obtained 30,846,354 transcripts sequenced, 30,303 differentially expressed genes from the control and stress treatment samples were identified, with 3363 genes expressed ≥ 2 and false discovery rate (FDR) of < 0.001. Six differential expression genes were then selected and qRT-PCR was used to confirm the RNA-seq results. Some of the genes (Include; *CML39, CBSX5, TRX1, GRXC9, SnRKγ1 and BAG6*) and signaling pathways discussed in this paper not been previously studied in quinoa. Genes with ≥ 2 were used to design the gene interaction network using Cytoscape software, and AgriGO software and STRING database were used for gene ontology. The results led to the identification of 14 key genes involved in salt stress. The most effective hub genes involved in salt tolerance were the heat shock protein gene family. The transcription factors that showed a significant increase in expression under stress conditions mainly belonged to the WRKY, bZIP and MYB families. Ontology analysis of salt stress-responsive genes and hub genes revealed that metabolic pathways, binding, cellular processes and cellular anatomical entity are among the most effective processes involved in salt stress.

## Introduction

Salinity is recognized as one of the most important environmental limitation^1^, causing significant economic losses for farmers. Halophytes are one of the best germplasms in terms of Reactive oxygen species (ROS) detoxification and signaling^2^. Most metabolic pathways lead to the continuous production of ROS, which negatively affects biological molecules^1^. Quinoa (*Chenopodium quinoa* Willd., Amaranthaceae) is a facultative halophyte native to the Andes in Bolivia and Peru and is considered as an alternative to major crops to eliminate the present food shortages^3^. This plant has a strong root system and is highly resistant to a wide range of abiotic stresses^3^. Some varieties of quinoa can even grow at salinity concentrations higher than seawater^4^. Plant adaptation to salinity stress through anatomical and physiological changes resulting from primary salt stress signaling pathways of salinity stress such as ROS, Ca^+2^ diffusion and phospholipid signaling^5,6^. Despite extensive information on the mechanisms of salinity tolerance in quinoa, studies on this topic are still limited to the transcriptional level^3,7^.

Ca^+2^ signaling is received by various proteins such as calcineurin B-like protein (CBL), Calmodulin (CaM), calmodulin-like proteins (CML) and calcineurin B-like protein-interacting protein kinase (CIPK), which in turn regulate the downstream targets and consequently, release the signaling cascade^8,9^. Analysis of the gene expression profile analysis showed that CMLs play a key role in the response to abiotic stresses such as drought and salinity^10,11^. Until now, CaMs and CBLs are the sole representatives of sensor relay proteins which transduce the signals via molecular interactions after binding to the second Ca^+2^ messenger^12^. It is suggested that Cystathionine b-synthase (CBS) proteins maintain cell redox homeostasis and regulate plant growth with the help of Thioredoxin (TRX) systems directly in Ferredoxin-Trx system (FTS) and NADP-TRX system (NTS), which consequently leads to the control of H_2_O_2_ levels^13^. glutaredoxins (GRXs)are placed into TRX superfamily along with thioredoxins. They are members of a multigene family of proteins and are considered as maintenance and regulatory mechanisms^14,15^. Maintenance of redox balance in the cell is critical for various signaling pathways and metabolic activities and is done by different isoforms of GRX^14^. Some protein kinases in the cell can sense the production or induction of ROS and respond to the stress via a series of phosphorylation and dephosphorylation signals. For example, NADPH oxidase can rapidly increase intracellular ROS levels and the induced signal is received by the nucleus through the plasma membrane^16^. Two protein kinases (SnRK2.4 and SnRK2.10) are released by SNF-related Kinase 1 (SNF1), the key component of cell cellular signaling network, to regulate ROS homeostasis and response to salinity stress in Arabidopsis^17^.

Calcium signaling plays directly regulates programmed cell death (PCD) by protein folding through chaperones. Bcl-2-associated athanogene (BAG) protein includes a BAG domain which binds to heat shock cognate protein 70 (HSC70) and a specific IQ motif which binds to the free cytosolic Ca^+2^ and acts as a mediating molecule to bind HSP70/HSC70 to the target protein^18^. Results of qRT-PCR analyses showed that *At*BAG6 transcription level was significantly upregulated by abiotic stresses such as salinity^19,20^. Also, three (AtBAG5,6 and 7) out of seven BAG proteins identified in arabidopsis have distinctive properties unique to plant BAG proteins which are probably regulated by calmodulin and Ca^+2^^21^. BAG family proteins are involved in various cell processes such as apoptosis, proliferation, differentiation and stress signaling^21^. Most molecular chaperones are stress proteins that exist as HSPs that strongly protect the cell against from injury such as salinity stress^22^. For example, the expression of 9 genes of *HSP* family increased under salinity stress in rice^23^.

Most studies on the effects of salinity on halophytes have been conducted with NaCl.This approach does not provide comprehensive information about the tolerance potential under field conditions, because the soil contains different salts that affect growth and germination^24^. Seawater is a mixture of saline solutions, similar to saline soils, and their synergism can affect seed germination^25^. Due to the potential of quinoa to grow under adverse conditions, sowing this plant in regions with saline water sources may be a good option.

The development of high-throughput sequencing technologies has allowed researchers to use the RNA-seq method to identify and compare the pattern of genes that affect salt tolerance and ultimately to describe the molecular mechanisms of tolerance in plants. Network analysis is an effective method for combining experimental data obtained from different molecular levels with all available molecular data^26^. This method can help to investigate the identified candidate genes identified from transcriptomic studies in a molecular interaction network. In this view, the clusters of this interaction network with the highest relationship between the candidate genes are identified as the major biological processes involved in a given study. This issue makes it possible to have a more holistic view about the studied process^26^. Quinoa is a potential crop in the middle east, northern Africa and central Asia, which have saline soil and water and thus struggle with limitations in crop production. To fully exploit the potential of quinoa as a suitable crop in marginal environments, identification and introduction of new high-yielding genotypes and by finding key genes or introduction of genes to current high-yielding cultivars is necessary, and our paper may help in this regard. The aim of this study was to use RNA sequencing and network analysis in the quinoa plant to investigate the genes involved in dealing with salt stress management and also to identify the effective hub genes, with the help of RNA sequencing and network analysis in quinoa plant.

## Results

According to the results of biochemical and molecular analyses such as superoxide dismutase, peroxidase, polyphenol oxidase, catalase, proline, glycin-betaine (data not shown), among the treatments 6 h, 1d, 2d, 3d, 4d, 5d, 6d and 7d after salinity stress at 6.9 dsm^−1^ and 13.8 dsm^−1^, 4d treatment at 13.8 dsm^−1^ was selected along with the control plant.

### Transcriptome sequencing and mapping

Response to salinity stress in quinoa plant cv. Titicaca was investigated using the RNA-seq technique. Among the total reads, 31,676,929 transcripts for control and 30,800,872 transcripts for 4 days after salinity stress treatment were specifically mapped to the reference genome using Star Aligner. The average length of the mapped reads was 197 bp (Table 1).

**Table 1:** Summary of Chenopodium quinoa, control read mapping against 4d (13.8 dsm^−1^) genes.

	(Treatment) time	
	Control	4d (13.8 dsm^−1^)
Number of reads	37,929,780	38,896,874
Number of input reads after trim	36,543,047	36,386,890
Average input read length	200	200
Uniquely mapped reads number	31,676,929	30,800,872
Uniquely mapped reads %	86.68	84.65
Average mapped length	197.07	197.08
% of reads mapped to multiple loci	7.76	8.88

### Identification of DEGs

The R software was used to identify the DEGs under salinity stress conditions compared to the control. 3363 DEGs were identified using edgeR and TMM method based on FDR < 0.001 and Log 2 FC |2|. Out of these DEGs, 1609 and 1754 genes showed significant up- and down-regulation after exposure to salinity stress, respectively.

### DEGs and gene ontology analysis

**Figure 1:**
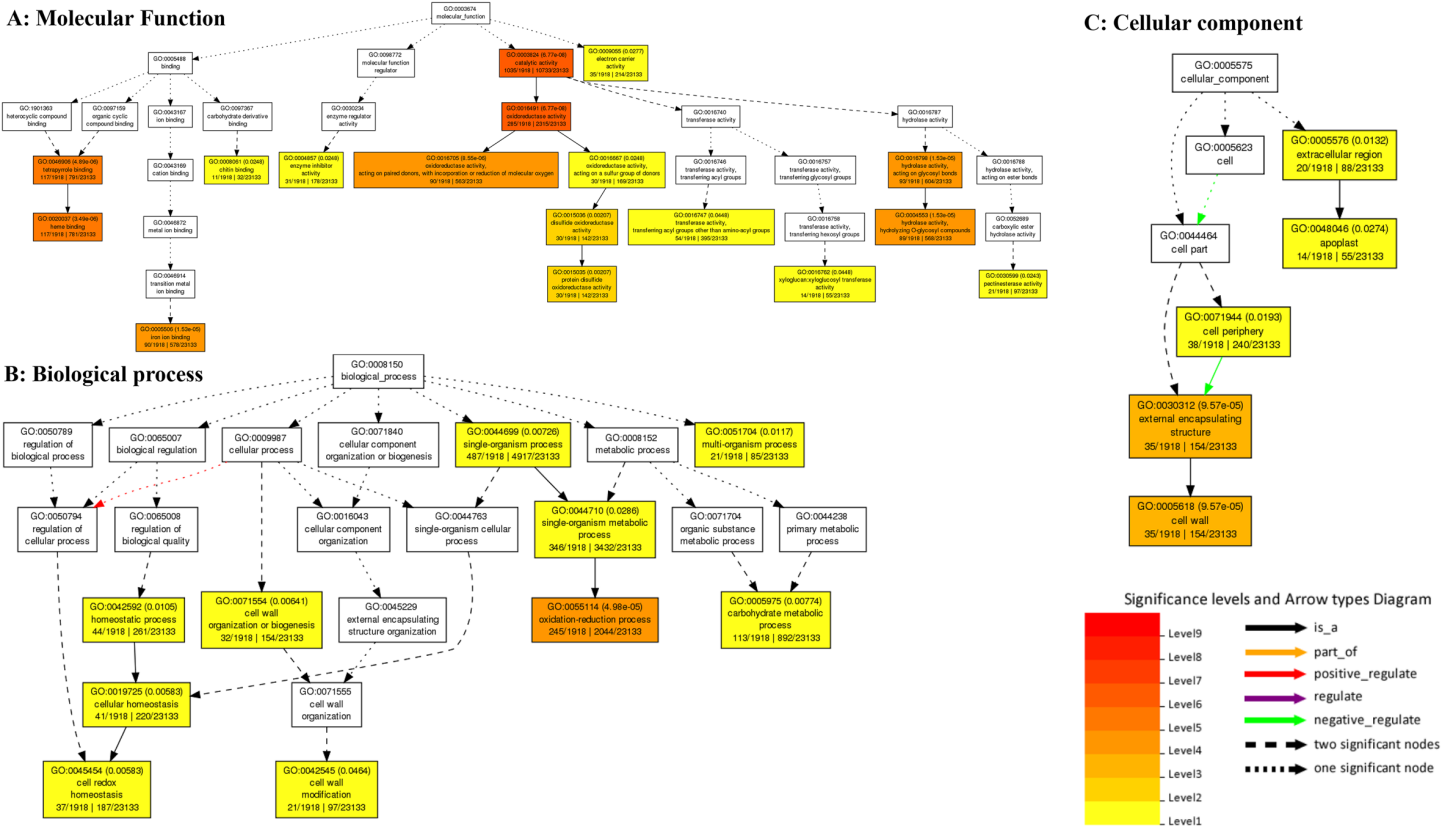
Pathway of the genes (increased and decreased expression in salinity treatment compared with control) under salinity stress conditions in quinoa based on three molecular processes.

**Figure 2:**
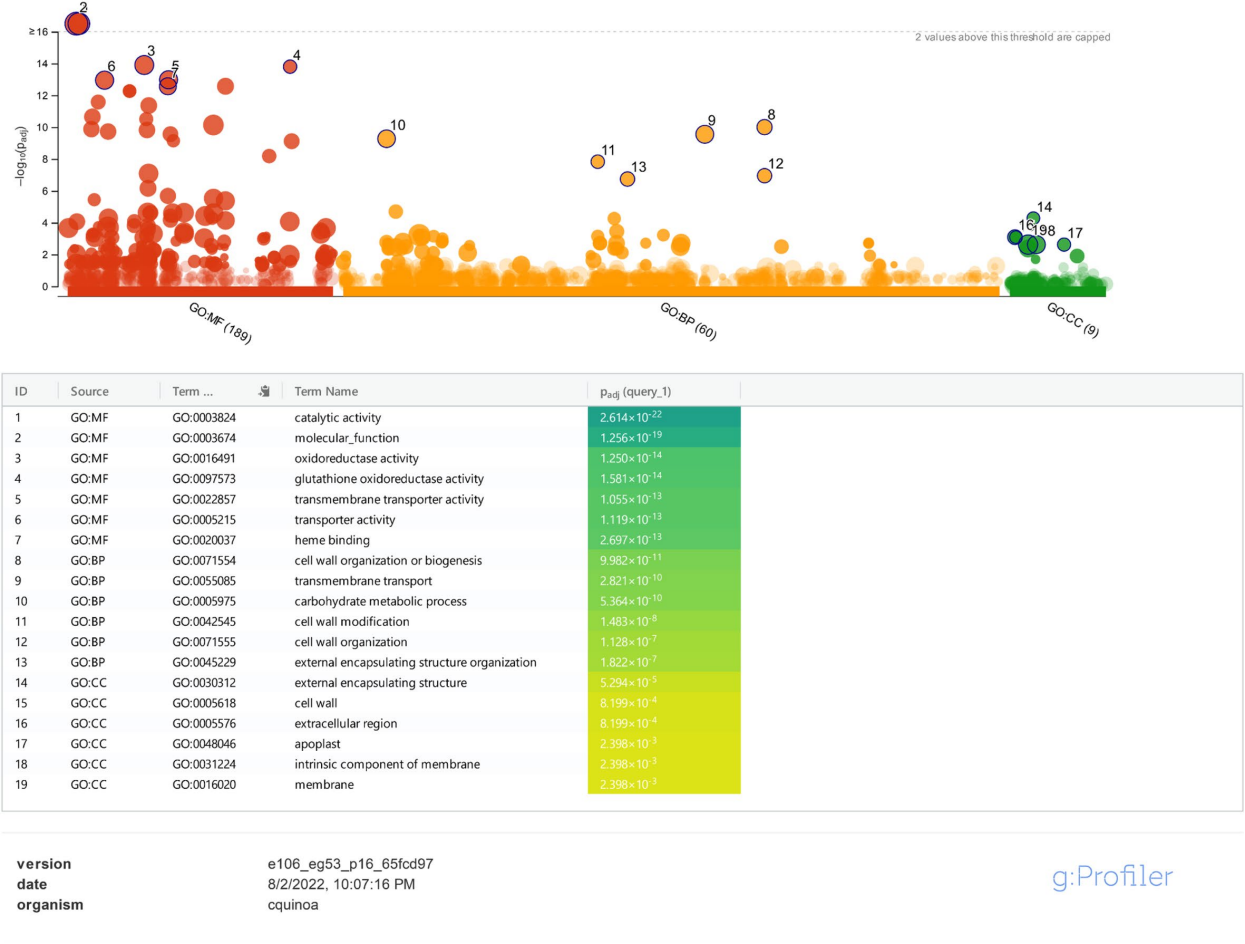
Ontology of the most important processes involved in salinity stress compared with control.

**Figure 3:**
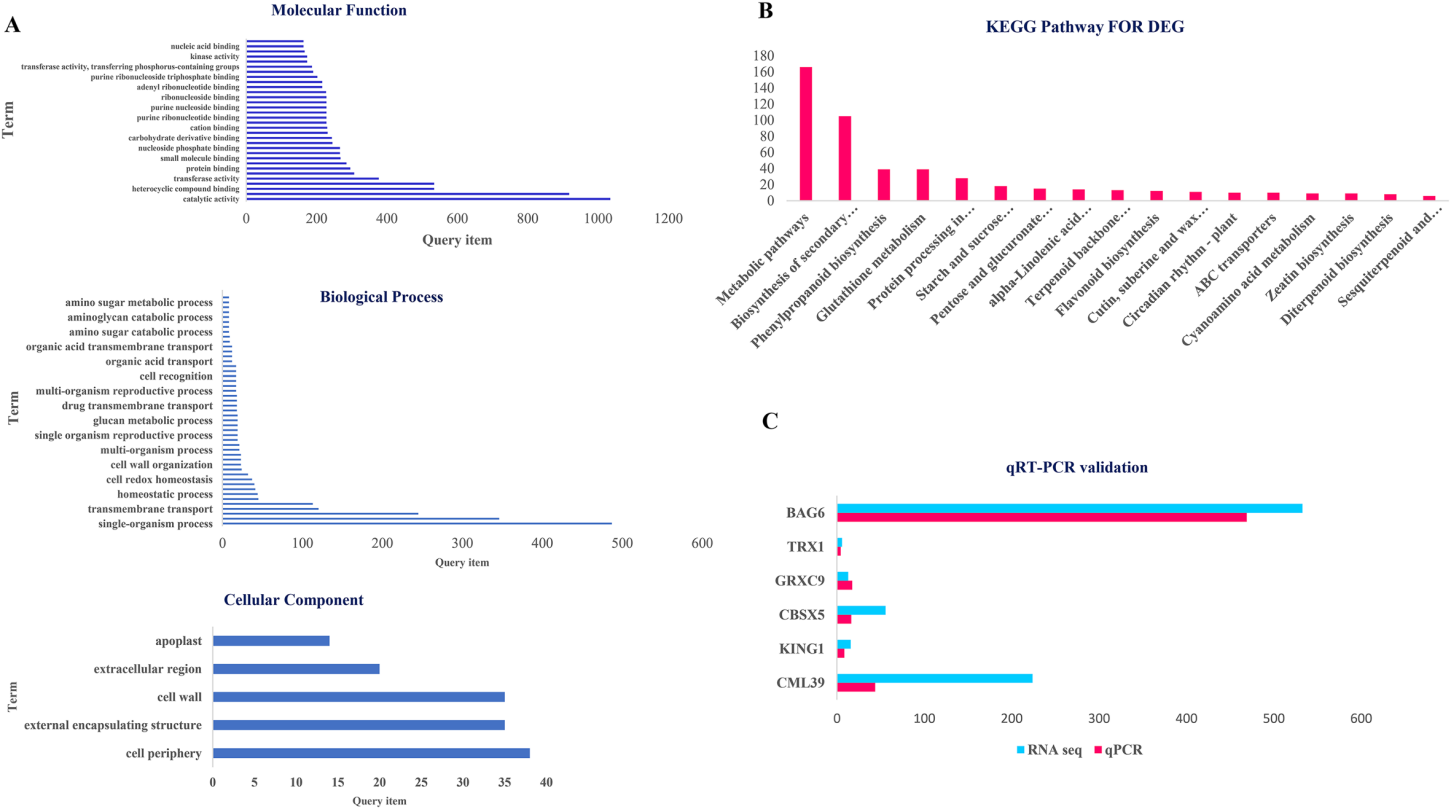
GO (**A**) and (**B**) DEGs- qRT PCR Validation (**C**).

DEGs were identified using Agri GO (Fig. 1) and g: Profiler (Fig. 2) and were classified into three main categories of molecular function, biological processes and cellular compartments (components) based on gene ontology analysis. Results showed that the highest number of genes involved in the molecular functions in salinity treatment compared to control were involved in catalytic activity and ‌oxidoreductase activity (Fig. 1a). Furthermore, DEGs involved in the biological processes in salinity treatment compared to control were related to transmembrane transport, oxidation–reduction process and carbohydrate metabolic process (Fig. 1b). Also, external encapsulating structure, cell wall and extracellular region pathways were involved in the cellular component category (Fig. 1c). On the other hand, catalytic activity had the highest frequency of transcripts involved in the molecular functions in salinity treatment. In addition, DEGs involved in the biological processes in salinity treatment included metabolic processes, single-organism metabolic processes and oxidation–reduction processes. In the cellular component, the cell periphery and external encapsulating structure pathways showed the highest number of transcripts (Fig. 3a). Also, the pathway obtained from the STRING database showed that the DEGs were mostly related to metabolic pathways and secondary metabolites biosynthesis (Fig. 3b).

### Validation of RNA-seq results using qRT-PCR

qRT-PCR was used to validate the RNA-seq results. Therefore, out of DEGs, six important genes involved in salinity that have not been previously studied in quinoa were selected from the DEGs, including *CML39*, *CBSX5*, *TRX1*, *GRXC9*, *SnRKγ1* and *BAG6*. According to the results, the expression of these genes in RNA-seq and qRT-PCR methods were consistent, and a validation rate of 94.44% was achieved (Fig. 3c).

### Validating the expression of selected genes using qRT-PCR

**Figure 4:**
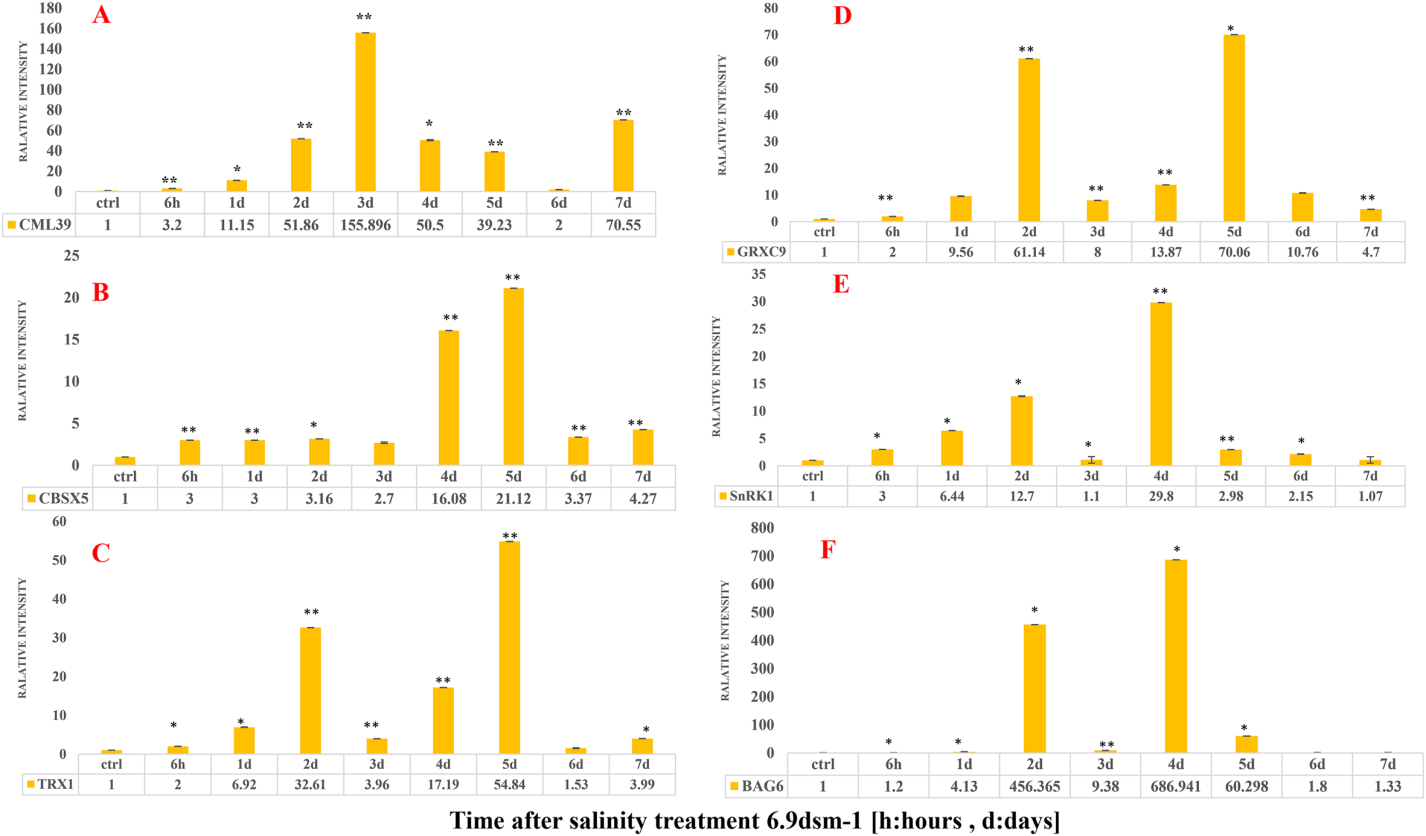
The changes in the expression level of CML39 (**A**), CBSX5 (**B**), TRX1 (**C**), GRXC9 (**D**), SNRK1 (**E**) and BAG6 (**F**) genes as affected by 6.9 dSm^−1^ salinity level over time.

**Figure 5:**
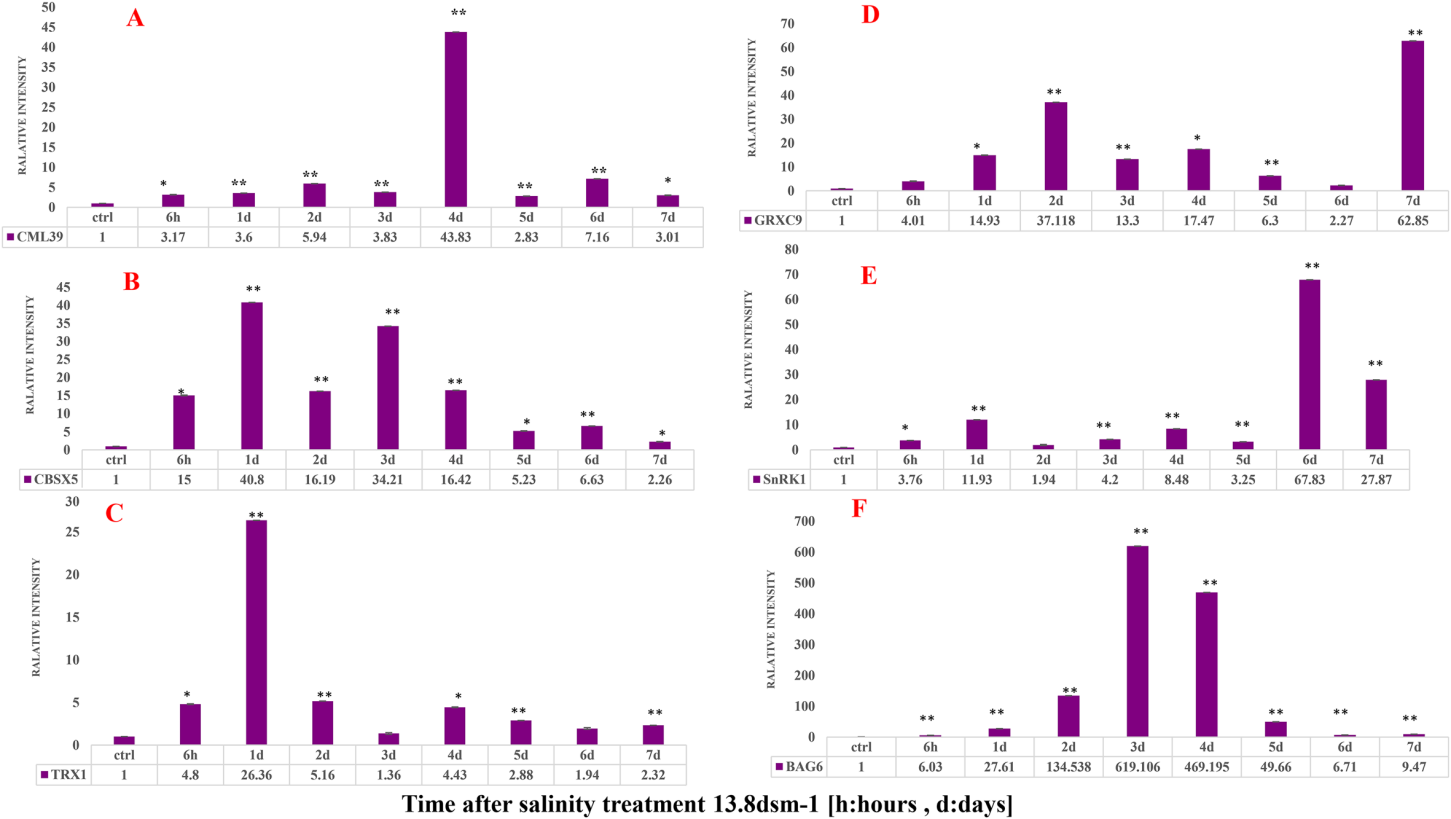
The changes in the expression level of CML39 (**A**), CBSX5 (**B**), TRX1 (**C**), GRXC9 (**D**), SNRK1 (**E**) and BAG6 (**F**) genes as affected by 13.8 dSm^−1^ salinity level over time.

The results showed an increase in *CML39* gene expression at 6.9 and 13.8 dSm^−1^ salinity levels compared to the control (Figs. 4a and 5a). At 6.9 dSm^−1^, this increase began 6 h after exposure to salinity and peaked on the day 3, then was bimodal until the end of the 7th day. At 13.8 dSm^−1^, this gene showed increased expression compared to the control on all days with the highest expression on the 4th day.

*CBSX5* gene expression increased in both treatments increased from the beginning of exposure to salinity (Figs. 4b and 5b). At 6.9 dSm^−1^, increased expression started on day 2 and reached its maximum on day 5. Also, the expression of this gene at 13.8 dSm^−1^ had a similar trend to that at 6.9 dSm^−1^.

*TRX1* gene expression increased immediately after the beginning of salinity stress exposure (Figs. 4c and 5c). At 6.9 dSm^−1^, the increase in expression started 6 h after salinity and peaked on day 5. The highest increase in the expression of this gene was related to the 1st day after stress at 13.8 dSm^−1^. Subsequently, gene expression increased and decreased inconsistently, and this decrease was not significant compared with the control.

An increase in *GRXC9* gene expression was observed at both salinity levels compared to the control (Figs. 4 and 5d). At 6.9 dSm^−1^, his gene showed a significant increase in expression on the 2nd day, with the highest increase on the 5th day. The highest increase in the expression of this gene at 13.8 dSm^−1^ was observed 2 and 7 days after salinity stress.

The results showed that the expression of *SnRKγ1* was increased compared to the control at both salinity levels (Figs. 4e and 5e). The peak of increased gene expression was observed on the 4th day at 6.9 dSm^−1^, whereas increased expression in the first hours of salt stress started at 13.8 dSm^−1^ and reached its peak on the 6th day.

*BAG6* expression was increased at both levels of salinity stress compared to the control (Figs. 4f and 5f). At both levels of salinity stress, the increase in expression started on the 1st day of stress exposure and reached its peak on the 4th day and then decreased.

### Network reconstruction

**Figure 6:**
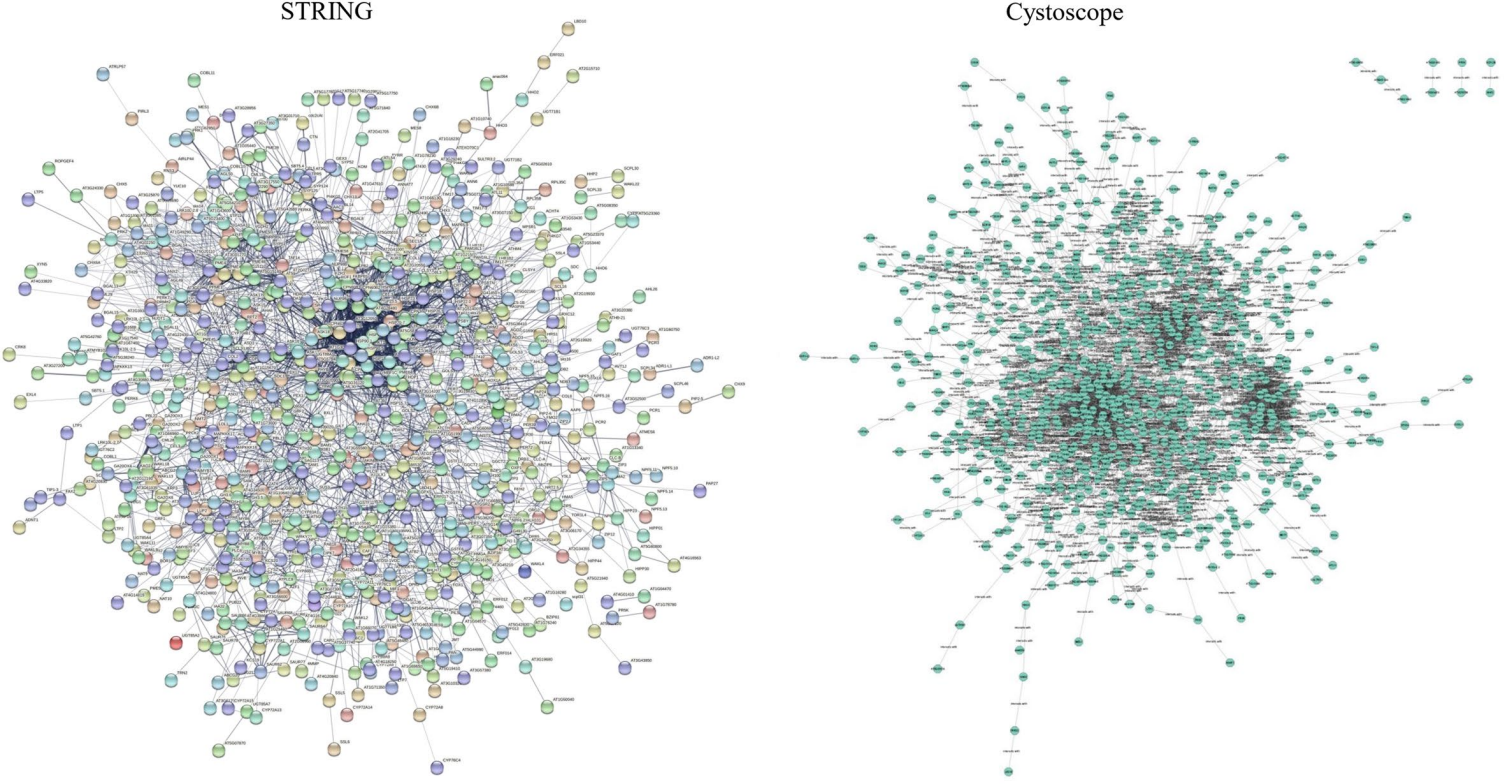
Cytoscape (right) and STRING (left).

**Figure 7:**
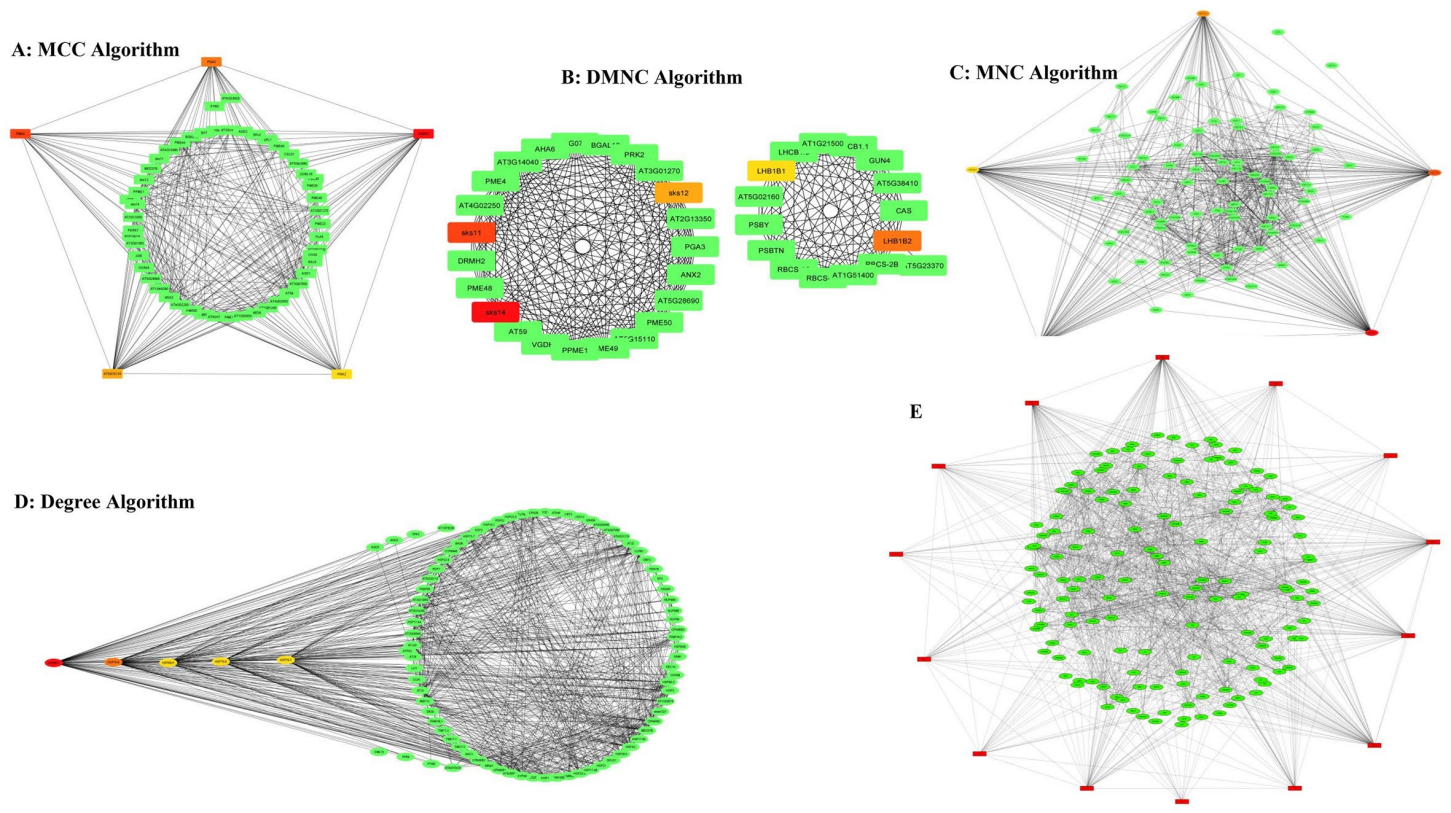
Gene interaction network for genes involved in response to salinity stress with differential expressions ≥ 2 using four different algorithms; a-d: four algorithms used to obtain hub genes, e: The network of related hub genes (specified on the vertex of each figure) drawn using Cytoscape software.

**Figure 8:**
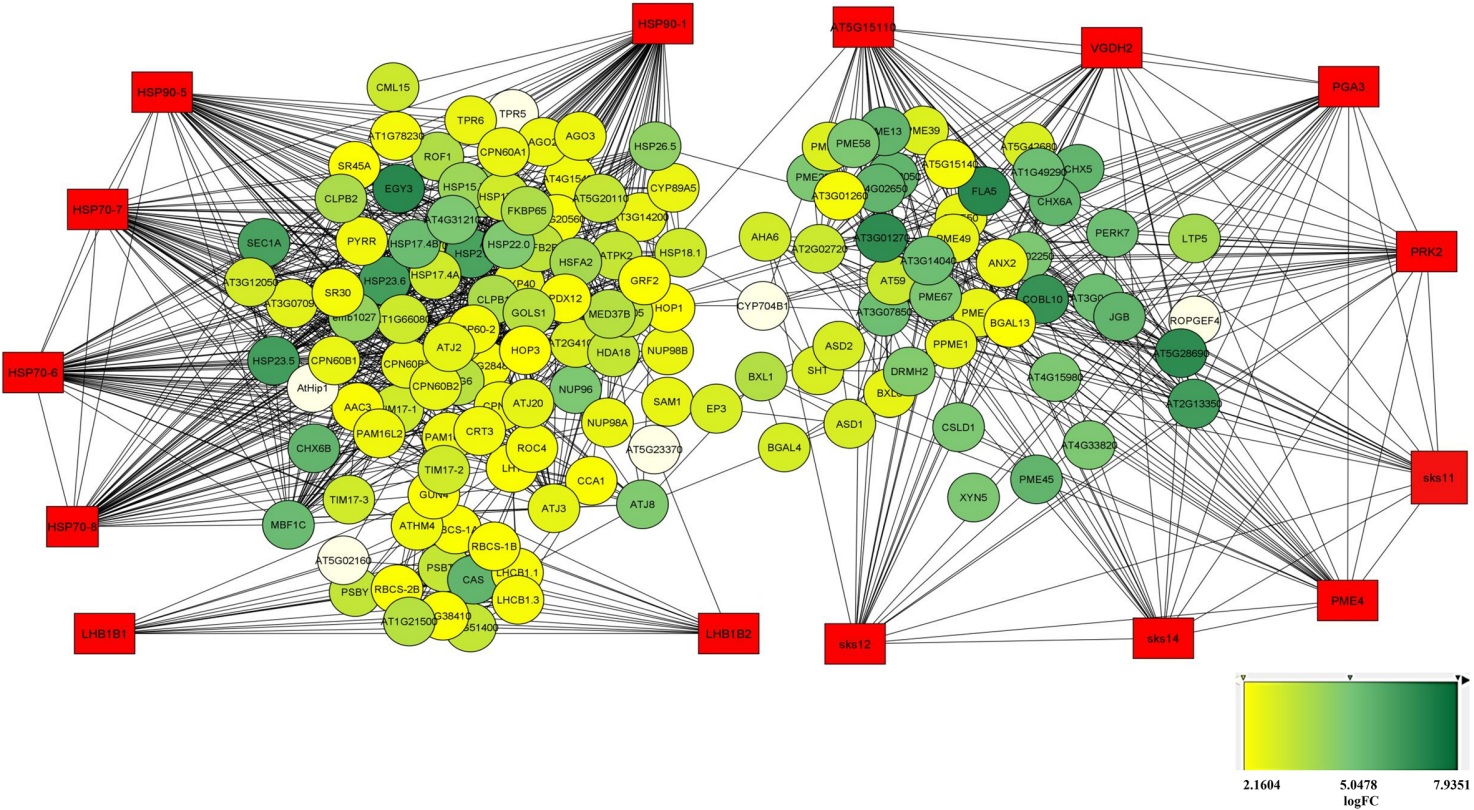
Gene relationship network of 161 genes with differential expressions ≥ 2 under salinity stress conditions and common neighbors.

The gene network of 842 resulted orthologue genes with expressions of ≥ 2 in response to salinity stress compared with control was drawn and visualized. Gene interaction network was drawn with 3116 protein interactions resulted from the STRING database (Fig. 6- left) using Cytoscape software (Fig. 6- right). Also, 14 genes with the highest interaction out of all these protein interactions were obtained using four algorithms (Fig. 7 and Table 2). Gene expression and regulation network was drawn based on the direct relationship among these 14 hub genes (Fig. 8).

**Table 2:** Hub genes with the highest interaction between DEGs (with FC ≥ 2).

Rank	Gene ID	Method	Gene description in Quinoa	FC	Quinoa	Arabidopsis Thaliana
1	VGDH2	MCC	Pectinesterase 4; PME4	5.279885	AUR62024556	AT3G62170
2	PME4	MCC	Pectinesterase 4; PME4	5.279885	AUR62024556	AT2G47030
3	PGA3	MCC	Exopolygalacturonase clone GBGE184; PGA3	4.583319	AUR62025531	AT1G02790
4	AT5G15110	MCC	Probable pectate lyase 3; AT59	6.839876	AUR62031638	AT5G15110
5	PRK2	MCC	Pollen receptor-like kinase 1; PRK1	5.279885	AUR62027982	AT2G07040
1	sks14	DMNC	At1g55570/T5A14_1; SKU5 similar 12	5.434871	AUR62006594	AT1G55560
2	SKS11	DMNC	At1g55570/T5A14_1; SKU5 similar 12	5.434871	AUR62006594	AT3G13390
3	LHB1B2	DMNC	Chlorophyll a-b binding protein 1, chloroplastic; LHCB1.3	2.299922	AUR62027587	AT2G34420
4	sks12	DMNC	At1g55570/T5A14_1; SKU5 similar 12	5.434871	AUR62006594	AT1G55570
5	LHB1B1	DMNC	Chlorophyll a-b binding protein 1, chloroplastic; LHCB1.3	2.299922	AUR62027587	AT2G34430
1	HSP90-1	MNC, Degree	Heat shock protein 90–1; HSP90-1	7.935131	AUR62031424	AT5G52640
2	HSP70-8	MNC, Degree	Heat shock 70 kDa protein 8; HSP70-8	3.798302	AUR62041322	AT2G32120
3	HSP70-6	MNC, Degree	Heat shock 70 kDa protein 7, chloroplastic; HSP70-7	6.919435	AUR62004581	AT4G24280
3	HSP70-7	MNC, Degree	Heat shock 70 kDa protein 7, chloroplastic; HSP70-7	6.919435	AUR62004581	AT5G49910
5,3	HSP90-5	MNC, Degree	Heat shock protein 90–5, chloroplastic; HSP90-5	2.500987	AUR62003042	AT2G04030

**Figure 9:**
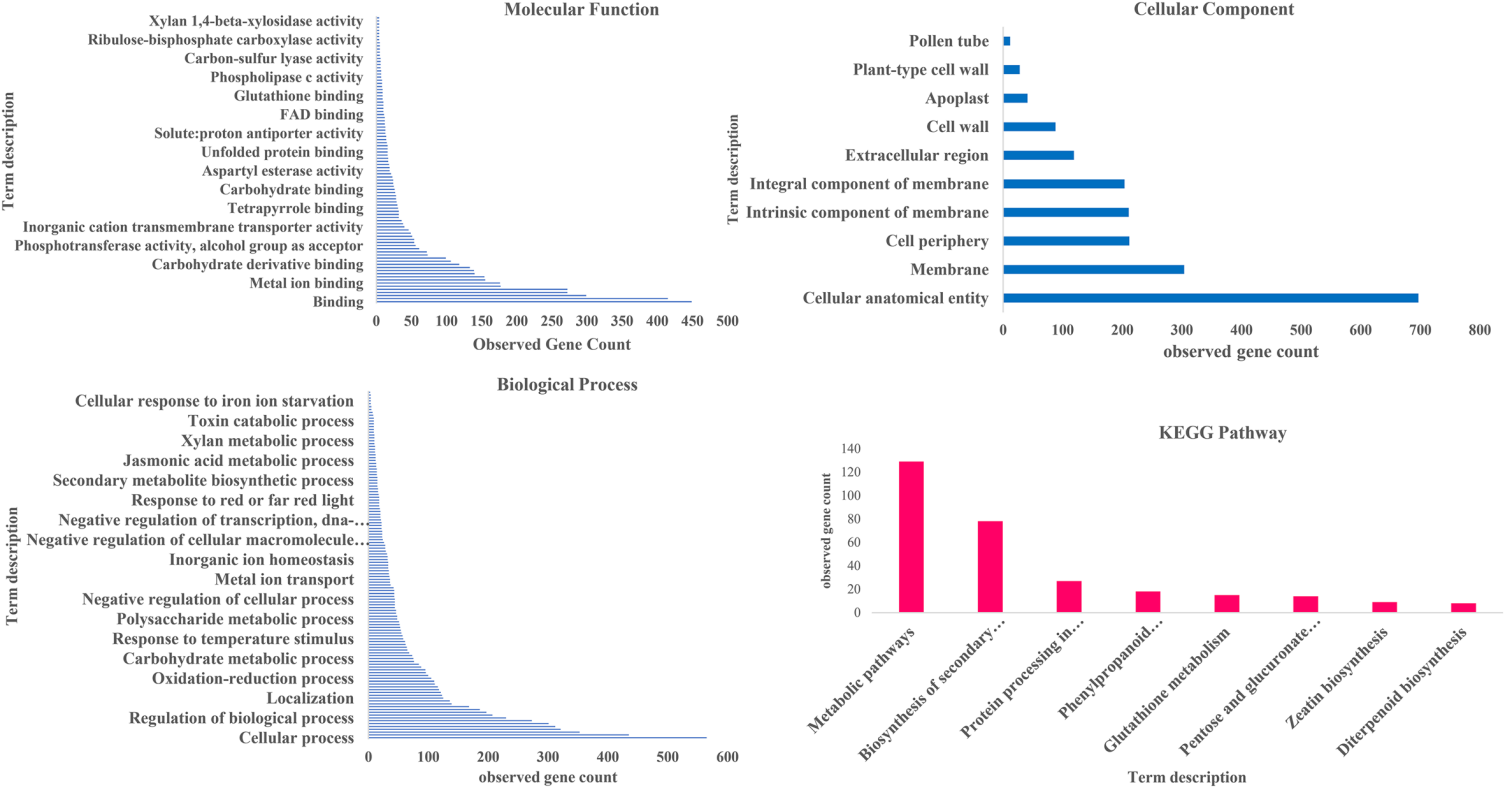
GO of the identified genes with differential expressions ≥ 2 in response to salinity stress.

**Figure 10:**
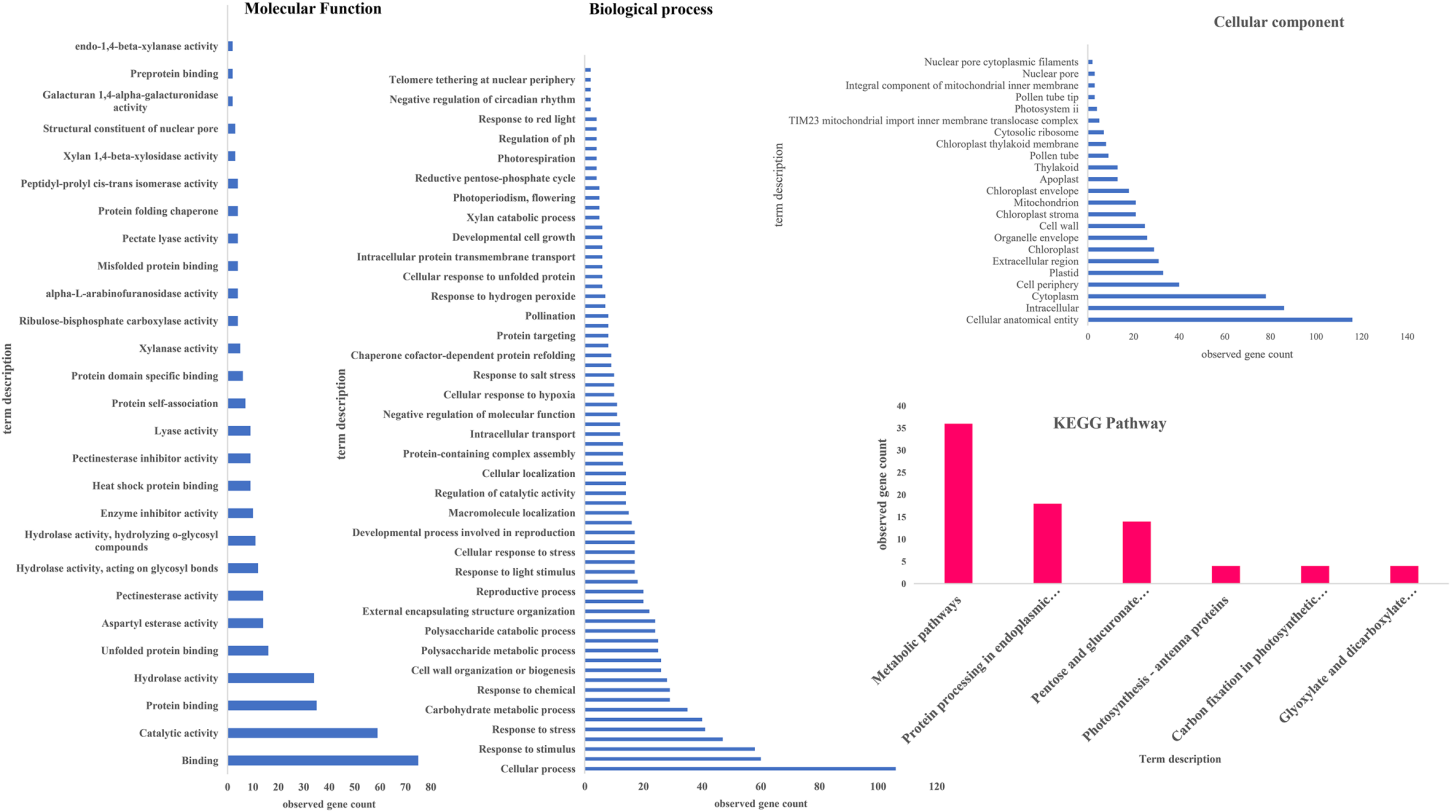
GO of the identified hub genes response to salinity stress.

Ontology of DEGs with FC ≥ 2 involved in salinity tolerance and hub genes was performed, and most genes involved in the molecular function, biological process and cellular component were respectively related to bindings, the cellular process and cellular anatomical entity. The most important identified pathways for DEGs with FC ≥ 2 resulting from STRING database were metabolic pathways and biosynthesis of secondary metabolites (Fig. 9), and in hub genes, metabolic pathway and protein processing in the endoplasmic reticulum pathway ranked first and second, respectively (Fig. 10).

**Figure 11:**
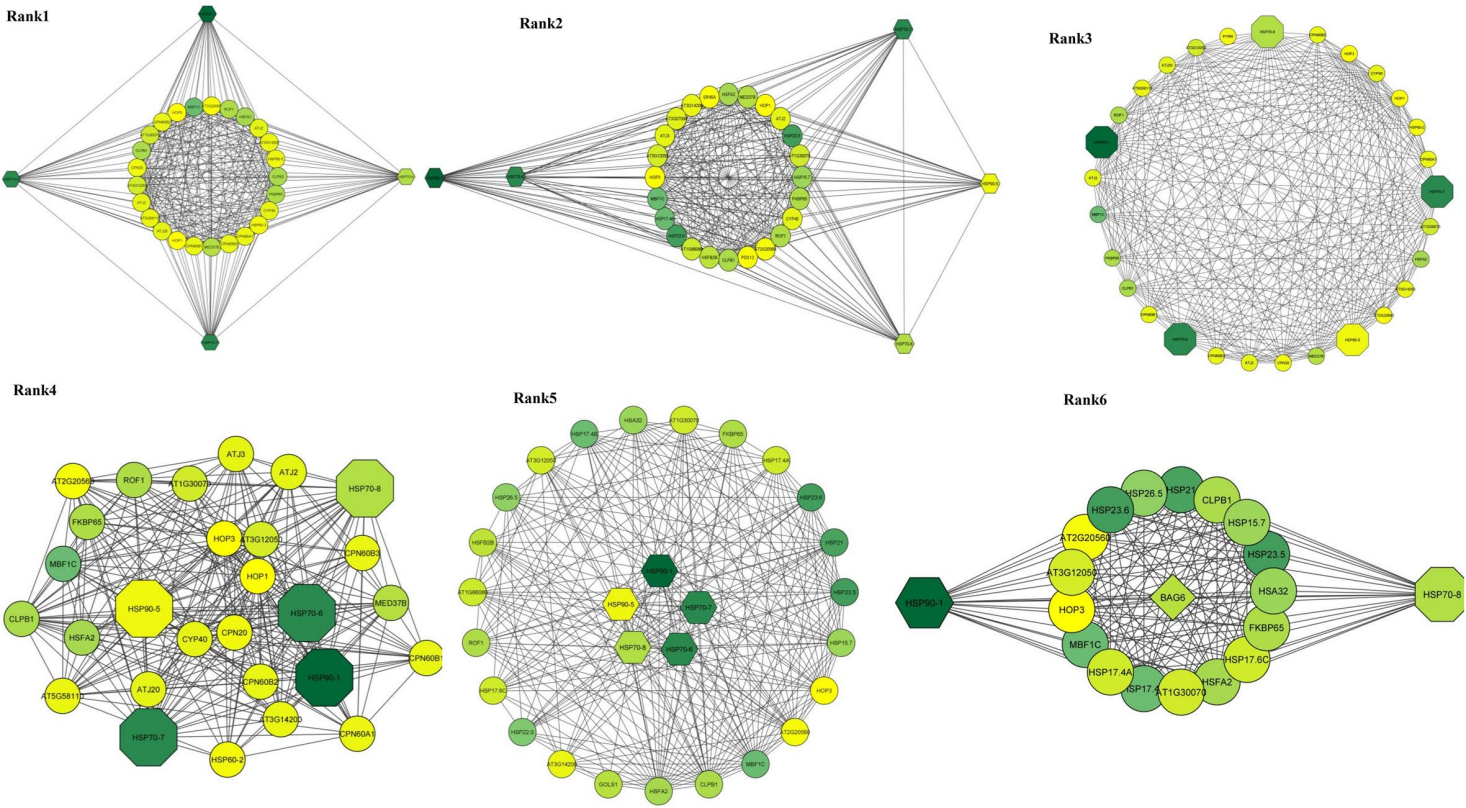
Six subnetworks with the highest interactions to find the most important hub genes.

This algorithm classifies the clusters based on the protein complex and is more applicable compared with the other algorithm of this plugin^27^. Out of 41 subnetworks resulting from the Cytocluster, the six with the highest rank which had the highest number of interactions and nodes, were selected (Fig. 11 and Table 3).

**Table 3:**
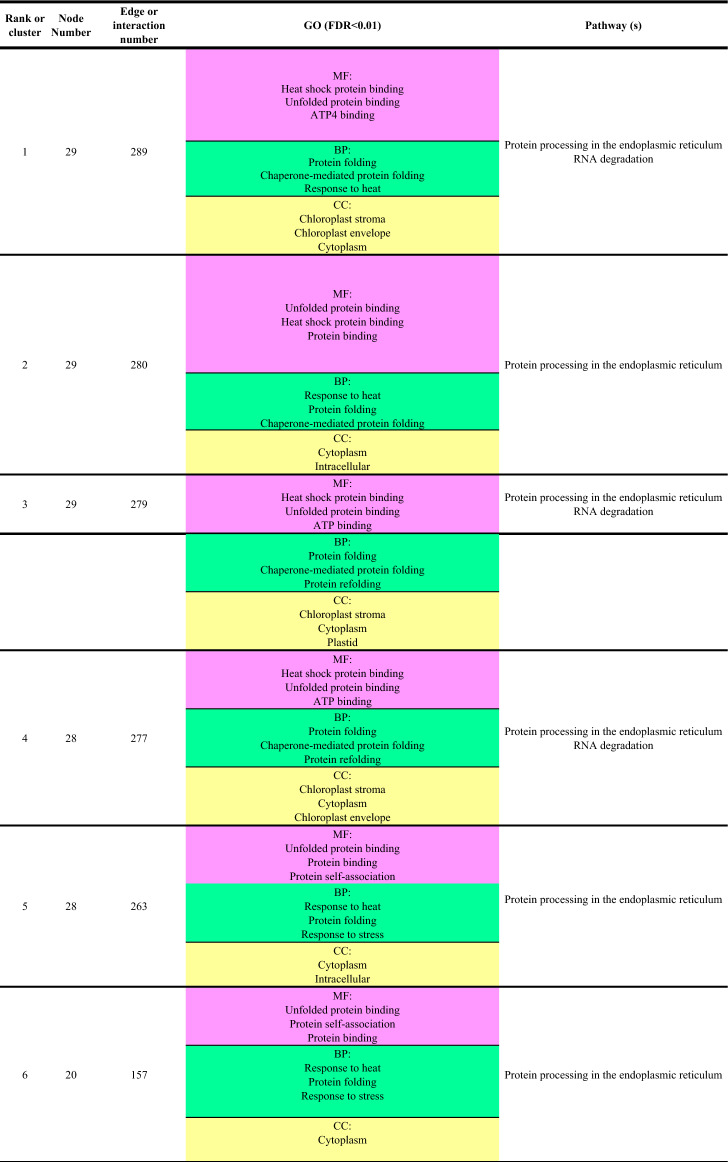
Six selected subnetworks with the highest rank regarding the highest number of interactions and nodes.

*MF* molecular function, *BP* biological process, *CC* cellular component.

Furthermore, out of all genes with different expressions, important families of transcription factors such as BZIPs, MYBs, NACs, WRKYs, C2H2 and BHLHs showed a high interaction with the identified hub genes (Table 4).

**Table 4:** Transcription factors involved in the expression of genes identified in response to salinity stress based on the analyses resulted from RNA-seq.

Gene	Gene ontology (GO)	Annotation	Fold change log2	FDR
AUR62039941	GO:0043565	Probable WRKY transcription factor 39;WRKY39	5.27	0.004
AUR62030160	GO:0043565	Probable WRKY transcription factor 35;WRKY35	6.37	0.01
AUR62021101	GO:0043565	Probable WRKY transcription factor 27;WRKY27	2.94	5.27 E−27
AUR62044535	GO:0043565	Probable WRKY transcription factor 75;WRKY75	2.37	0.007
AUR62021917	GO:0043565	WRKY transcription factor 42;WRKY42	−2.21	0.00
AUR62030596	GO:0043565	WRKY transcription factor 55;WRKY55	−2.24	4.184 E−07
AUR62023484	GO:0043565	Probable WRKY transcription factor 41;WRKY41	−0.39	6.62 E−220
AUR62001049	GO:0043565	Probable WRKY transcription factor 21;WRKY21	−2.45	5.11 E−31
AUR62009104	GO:0043565	Probable WRKY transcription factor 50;WRKY50	−2.75	2.50E−161
AUR62004778	GO:0003677	F12P19.8 protein;NAC028	4.92	0.01
AUR62003400	GO:0003677	F12P19.8 protein;NAC028	3.79	2.13 E−08
AUR62001365	GO:0003677	NAC transcription factor 47;NAC047	2.12	0.001
AUR62003842	GO:0003677	Myb family transcription factor EFM;EFM	11.82	4.84 E−172
AUR62023242	–	Transcription repressor MYB4;MYB4	5.27	0.007
AUR62040208	–	Myb/SANT-like DNA-binding domain protein;At4g02210	5.27	0.007
AUR62014702	–	Transcription factor MYB102;MYB102	4.92	0.01
AUR62036035	–	Transcription repressor MYB4;MYB4	4.92	0.019
AUR62022709	–	Transcription repressor MYB4;MYB4	2.32	1.54 E−25
AUR62002848	–	Transcription factor MYB62;MYB62	2.26	0.000
AUR62002136	–	Transcription factor MYB15;MYB15	2.21	1.25 E−37
AUR62003939	–	MYB transcription factor;C7A10.100	2.11	6.89 E−219
AUR62037317	–	Transcription factor MYB62;MYB62	2.09	0.006
AUR62043487	GO:0003677	Ethylene-responsive transcription factor ERF071;ERF071	6.48	0.000
AUR62018400	–	C2H2 and C2HC zinc fingers superfamily protein;At5g01860	2.21	0.000
AUR62032564	–	C2H2 and C2HC zinc fingers superfamily protein;At5g10970	−3.86	2.8 E−09
AUR62041399	GO:0006355	bZIP transcription factor 53;BZIP53	7.57	0.000
AUR62038993	GO:0006355	Basic leucine zipper 61;BZIP61	3.67	0.000
AUR62004028	–	BZIP transcription factor-like protein;bZIP7	2.19	1.35 E−24
AUR62003771	–	BZIP transcription factor, putative (DUF630 and DUF632);At4g39790	2.04	0.03
AUR62005699	GO:0046983	Transcription factor bHLH49;BHLH49	8.58	3.87 E−19
AUR62031976	GO:0046983	Transcription factor bHLH87;BHLH87	6.65	3.47 E−38
AUR62000871	GO:0046983	Transcription factor bHLH96;BHLH96	5.27	0.007
AUR62018701	GO:0046983	Transcription factor bHLH94; BHLH94	5.27	0.007
AUR62038707	GO:0046983	Transcription factor bHLH85; BHLH85	4.92	0.01
AUR62028228	–	Transcription factor bHLH104; BHLH104	4.92	0.01
AUR62016073	GO:0003677	Transcription factor bHLH125;BHLH125	2.51	0.001
AUR62003664	–	Transcription factor bHLH131;BHLH131	2.51	0.02
AUR62003654	GO:0046983	Transcription factor bHLH30;BHLH30	2.42	9.55 E−14
AUR62020904	GO:0046983	Transcription factor bHLH78;BHLH78	2.27	2.67 E−280
AUR62014828	GO:0006357	Transcription factor bHLH162;BHLH162	−2.22	0.001
AUR62021282	GO:0006357	Transcription factor bHLH36;BHLH36	−2.42	1.01 E−09
AUR62033947	GO:0046983	Putative transcription factor bHLH041;BHLH41	−2.86	1.31 E−11
AUR62004073	GO:0046983	Transcription factor bHLH25;BHLH25	−2.94	9.38 E−29
AUR62028866	GO:0046983	Transcription factor bHLH66;BHLH66	−3.25	1.03 E−19
AUR62029052	GO:0046983	Putative transcription factor bHLH041;BHLH41	−4.20	0.00

## Discussion

In this study, we first identified genes that showed significant differences in salt stress response compared with the control, most of which followed the metabolic pathways. Among the resulting DEGs, six DEGs were investigated for the first time. the gene interaction network was than reconstructed to dine the most important DEGs and identify hub genes, among which the heat shock family was observed the most. Finally, protein processing in the endoplasmic reticulum was identified by ontology analysis as one of the most important pathways responding to salinity stress in quinoa.

Previous studies have shown the overexpression of *CML* genes under salinity and cold stresses^28^ which are promising candidates to obtain plants with improved abiotic stress tolerance and respond physiologically to a wide range of stimulants received by plant cells^29^. Since the level of Ca^2+^ in cells is low under non-stress or low-stress conditions^30^, Ca^2+^ channels are temporarily opened to receive the signals, which leads to rapid entry of Ca^2+^ into cells. These changes are identified and coded by Ca^2+^ sensors such as CMLs^31^ which consequently regulate the downstream targets and activates the signaling cascade^9^. The kinase activity of this sensor results in the structure change and connection with the downstream target proteins^32^. In the present study, 23 *CML* encoding genes with different expressions were identified and one of the sensors- *CML39*- was investigated. In a similar study on *Arabidopsis thaliana*, 50 *CML*-encoding genes with different expressions were identified^10^. In this study, the results of qRT-PCR for the *CML39* gene showed that in general, the treatments in general compared to those given by control increased the expression of genes. Significant increase in the expression of *the CML39* gene due to salinity treatment with 6.9 dsm^−1^ started two days after salinity stress and peaked on the 3rd day. This peak occurred on the 4th day at 13.8 dsm^−1^ (Fig. 4). According to the results reported by McCormack et al.^10^ on the *CMLs* of *Arabidopsis thaliana* under drought and salinity conditions, *CML37*, *CML38*, *CML39* and *CML40* were among the highly upregulated genes under salinity stress conditions^32,33^. Results of qRT-PCR showed a significant increase in the expression of *CML39* 4 days after salinity stress, which was in accordance with the results of RNA-seq (Figs. 5a and 6a). Gene expression at two salinity levels revealed that the expression of this gene greatly increased under low-stress salinity. Activation of calcium sensors is regarded as a low-cost mechanism for the cell^34^. It is probable that due to the increased expression of this gene, the cell turns off the energy-consuming pathways and stimulates the functional genes via the expression of sensors to maintain the plant under stress conditions. However, when a plant is exposed to high levels of stress, these sensors interact with other related pathways and often activate a phosphorylation cascade, and target the main genes responding to stresses or transcription factors controlling these genes^34^. Products of these genes eventually lead to plant adaptation and help the plant to survive adverse conditions. Previous studies on genome-wide analysis showed that the transcription of *CML*s can be significantly affected by abiotic stimulants in tea, apple and grape^28,35^. Our results showed that *CML39* probably acts as a gene that responds to salinity stress in plants. Although the genes of the CML family are mostly unidentified in quinoa, this study shed light on the role of *CML39* gene expression against various levels of salinity stress. Gene ontology analysis showed that this gene is related to the calcium ion binding cluster and is considered a functional gene. Interestingly, according to the f RNA-seq results, we identified a group of genes that were co-expressed with the *CML39* gene (Table 4). For instance, WRKYs were associated with a cluster of TFs that were co-expressed with the *CMLs*. WRKY transcription factors are important regulators of signaling mechanisms that regulate various cellular processes for salinity tolerance^36^ Calmodulins and Calmodulin-likes have been shown to interact with MAP kinases to regulate transcription and reprogramming^37^.

In plants, *CBSXs* sense the changes in ion and energy balance and transfer the information to various plant organelles, which helps to maintain ion and energy homeostasis and consequently, results in a better tolerance against abiotic stresses such as salinity. For example, it is reported that *CBSX4* may play a critical role in salinity tolerance in *Oryza sativa*^38^. Furthermore, *CBSX4* is shown to be a stress-related gene and its overexpression in tobacco leads to increased tolerance against abiotic stresses. Our results also showed an increase in the expression of this gene against various levels of stress (Figs. 5c and 6c). *CBSX* proteins which have only one pair of CBS domains, are directly involved in the activation of TRXs and thus, regulate the levels of cell H_2_O_2_ and regulate plant growth and development (Figs. 5c and 6c). The Results of the present study indicate that at both levels of salinity stress, the increased expression of the *CBSX5* gene coincided with the increase in the expression of the *TRX1*gene due to the regulation of *TRXs* by *CBSX*. This increased expression probably controls the level of ROS in the cell and thus has a positive effect on plant growth under stress conditions. The Arabidopsis genome consists of six active *CBSX*s in different cellular components such as chloroplast (*CBSX1* and *CBSX2*), mitochondria (*CBSX3*), cytosol (*CBSX4*) and endoplasmic reticulum (*CBSX5* and *CBSX6*) and it can be safely assumed that *CBSX* is required for a sensor relay protein such as CaM and CBL^13^. Furthermore, proteins possessing the CBS domain are found in all kingdoms of life except for viruses, thus far. For instance, the number of proteins containing the CBS domain identified in *E. coli*, *Saccharomyces cerevisiae*, *Arabidopsis Thaliana*, *Oryza Sativa* and *Homo Sapiens* is 8, 12, 34, 59 and 75, respectively^39,40^. Results of RNA-seq led to the identification of 9 proteins that contained the CBS domain in quinoa. Pathways obtained from the STRING database also indicated the activity of this gene in the metabolic pathways.

In living cells, ROS play a key role in signal transduction. However, these compounds also damage the macromolecules. The concentration of ROS in mitochondria- as well as in other compartments- must be strictly controlled. Plant mitochondria contains several antioxidant systems that can repair the damage to macromolecules and probably act as redox sensors. These include glutathione-dependent pathways and systems based on glutaredoxin (GRX) and thioredoxin-like (TRX) molecules. In one experiment, the transfering of the *GRX* gene to Arabidopsis resulted in improved cold tolerance in the plant^41^. overexpression of the *GRX* gene under heat (45 °C), cold (4 °C) and saline (150 mM NaCl) conditions also indicated tolerance in *Oryza Sativa*^42^. Investigation of gene expression profile in Arabidopsis in response to various biotic and abiotic stresses showed that the *GRX* and *TRX* genes play a key role in stress tolerance^43^. The number of *TRX* genes in plant species may vary from 11 in sorghum to 60–70 in rice and Arabidopsis^44^. In the present study, RNA-seq results revealed that the total number of *TRX*s and *GRX*s identified in quinoa was 19 and 51, respectively. It seems that cold stress decreases the expression of most *TRX* genes, but drought stress -at least in the early stages of stress- leads to the upregulation of this gene^45^. Since *GRX*s are members of the *TRX* family, the interaction of these genes leads to upregulation of *CBSX5* and helps to improve stress tolerance by maintaining the balance and control of H_2_O_2_ in the cell. Previous studies have shown the critical role of *GRX*s in the tolerance to abiotic stresses such as oxidative stress and metals^46^. In a study conducted by Kumar et al.^47^ on two cultivars of chickpea (*Cicer arietinum* L.), the *CaGRX* gene was investigated for overexpression and various biochemical and physiological parameters related to salinity and drought stresses. The results showed that *CaGrx* improved the plant tolerance to drought and salinity by positive regulation of the antioxidant defense system and different stress-related parameters. Increased *CaGrx* expression improve plant biochemical and physiological performance in response to drought and salinity stress by activating the antioxidant defense system. The decrease in ROS levels under high salinity and drought conditions may be due to the overexpression of *CaGrx*, which increases catalase and *APX* activity and directly decreases H_2_O_2_ levels and DHA expression^48^, which is in accordance with the results of the present study. As a defense mechanism, *GRX* limits excessive ROS production, participates in redox signaling, and directly or indirectly enhances antioxidant defense mechanisms^47^. Gene ontology of the *TRX1* and *GRXC9* genes showed that these genes are related to the process of organic substance metabolism, cell process single organism process and biological regulation, involved in the biological processes.

The SnRKs family is divided into SnRK1, SnRK2 and SnRK3 subfamilies. It is established that most members of the SnRKs family play essential roles in response to abiotic stresses. SnRKs cooperate with TFs in the maintenance of cellular energy balance^49^. Previous studies have shown that TFs are activated simultaneously with protein kinases, which act as signal transmitter/receiver proteins in the membrane^50^. These TFs included the *bZIP*, *C2H2*, *BHLHs*, *ERF*, *MYB*, *NAC* and *WRKY* families and each had a different expression in salinity tolerance in plants, which is in accordance with the results of Arisha et al.^50^ According to the RNA-seq results in the present study. All of these TFs had specific and significant expressions 4 days after salinity stress treatment, which indicates the mechanisms of stress tolerance (Table 4). Since SnRKs belong to late genes, qRT-PCR results showed that the expression level was significantly increased compared to the control at 13.8 dsm^−1^ in the last days of stress. These genes were slowly activated within hours after stress and often showed a stable levels of expression. Transcription factors such as early genes activate and encode the most important genes that respond to stress (delayed genes).

Szymańska et al.^17^ found out that *SnRK2.4* and *SnRK2.10* in cooperation with *SnRK1* can maintain the ROS homeostasis and response to salinity stress in Arabidopsis^17^. SnRK1 acts as a key kinase in stress response, and overexpression of *PpSnRK1α* can significantly improve salinity tolerance by regulating ROS metabolism regulation or ABA-mediated pathways. It has been reported that the overexpression of the gene encoding the α-subunit of SnRK1 in *Prunus persica* (*PpSnRK1α*) can enhance salinity tolerance. Overexpression of *SnRKγ1* led to lower leaf damage, increased proline, and decreased malondialdehyde (MDA) content compared with the control under salinity stress conditions (data now shown), which was similar to the results of^49^. The results of qRT-PCR for the *SnRKγ1* gene showed that the expression of the gene in the plant increased during the days after the stress. A significant increase at 6.9 dsm^−1^ began on the 3rd day and peaked after 4 days of exposure. In fact, when the plant is affected by ROSs, the expression of this gene at high levels neutralizes the effects of ROSs and prevents the induction of stress^7^. This also occurred at 13.8 dsm^−1^ salinity level, and since the plant had to cope with more salt content at this level, increased expression started from the 1st day and continued until the end. It may be concluded that the plant utilized the mechanism of increased SnRKγ1 expression to prevent the excessive consumption of ATP as well as to control the adverse effects of ROSs.

The BAG family recruit molecular chaperones using their domains under stress conditions to target proteins and change their function by altering the protein conformationBAG proteins regulate various physiological processes such as apoptosis, tumor induction, stress response and cell cycle. *BAGs* also regulate HSP chaperone proteins (positively or negatively) and form complexes with various transcription factors^51^. At the transcriptional level, *BAG* family genes in plants have key roles in the PCD processes which range from growth, and tolerance to fungi to abiotic stress tolerance^52^. The results of the expression of this gene at 6.9 dsm^−1^ salinity level showed that from the early days of stress, the plant increased the expression of *the BAG6* gene to prevent the stress, so that on the 2nd day, cell apoptosis and PCD were prevented by overexpression and with the help of chaperones. This reached it is peak 4 days after stress in 13.8 dsm^−1^ treatment. It seems that the damage inflicted upon the plant has irreversible effects which leads to high energy consumption. However, the cooperation between the BAG family and chaperones will help the plant to maintain its balance under adverse conditions. Under abiotic stress, a strong induction of the genes in the *BAG* family has been observed^21^. Also, it is demonstrated that ABA is involved in the regulation of *BAG* gene in *Arabidopsis thaliana* and plays a critical role in response to abiotic stresses^21^. GO results for the *BAG6* gene showed that this gene is associated with chaperone binding and protein binding category and most interact with chaperones and binding proteins to mitigate the stress through a significant increase in the expression level (Table 3, Rank6).

To better understand the mechanisms involved in salinity tolerance, network analysis was done to identify the hub genes and their associated gene out of thousands of genes involved, which led to the identification of subnetworks that covered the highest number of hub genes with the lowest number of edges. In general, out of these genes and based on the used algorithm and plugin, 161 genes with a different increase in expression were identified, out of which 14 genes were nominated as hubs. These 161 genes may be specifically involved in response to salinity stress. Gene ontology obtained from this cluster showed that most genes were placed in metabolic activity, protein processing in the endoplasmic reticulum and pentose and glucuronate interconversions pathways, respectively. These results were obtained by covering only 14 hub genes with Log2 FC > 2. The HSP family, including HSP90 played an important role in this stress and regulated numerous transcription factors such as *WRKYs*, *bZIPs* and *MYBs* through interactions with other genes.

Some plants use the induction of heat shock genes as a mechanism to for cell survival under stress conditions^53^. According to the results of the network analysis in the present study, out of 14 resulted hubs, the most important hub genes were related to the HSP family, specifically *HSP90*. The *HSP90* subfamily is an ATP-dependent molecular chaperone with a highly conserved sequence in the bacteria and eukaryotes and homologs found in different organisms. In fungi and animals, this subfamily plays an essential role in sending stress signal such as the folding of steroid hormone receptors, protein kinases, transcription factors and substrate activation to start sending stress signal^54^. Recent studies on the plant *HSP90* subfamily is mostly focused on evolution analysis and physiological performance^55^. In most plants, some of the genes from this family have been identified that are expressed immensely under salinity, heat, drought and heavy metal stresses^56^. The *HSP* gene family has various functions in plants and is involved in a wide range of biological processes, especially in the response to abiotic stress^57,58^. In another studies, the expression of *OsHSP* genes increased under salinity stress conditions^59^.

According to the results, the endoplasmic reticulum pathway was observed along with the increased expression of *the HSP90* subfamily in most hub genes, indicating that the endoplasmic reticulum play an important role in minimizing salinity stress. An increase of the *HSP90* protein in the endoplasmic reticulum can regulate the changes and targeting of the vacuole and plasma membrane ion transporters by reducing cytosolic sodium to confront salinity stress^60^. In addition, an increase in the *HSP90* protein-especially in chloroplasts or endoplasmic reticulum- can lead to general homeostasis, or increase salt and osmotic stress tolerance by altering organelle input–output system or protein homeostasis. This protein is critical for the homeostasis of stress tolerance proteins and response to stress. Therefore, besides being induced in response to short-term abiotic stresses, their production is an essential stage in plant adaptability to abiotic stresses^57^. *HSP90* is critical in protein folding and is involved in signal sending pathways, protein degradation and their movement^61^. They also bind to a chaperone named HSP70 in many complexes. It seems that among the genes that were chosen as hubs, the role of HSP90 is more pronounced compared with the other genes. To ensure that whether the HSP family members are the most important genes among the 14 identified hubs, the Cytocluster plugin and IPCA algorithm were used. It was revealed that the HSP family, especially HSP90 and HSP70 directly interacted with the BAG6 gene, which is among the genes investigated in the present study (Fig. 11, rank 6). This interaction helps to maintain the internal conditions of the plant against high levels of salinity which lead to ROS production^62^. In the present study, this interaction probably prevents cell apoptosis and PCD in quinoa. Besides osmolites, chaperones of the HSP90 and HSP70 families and their companions interact with numerous signaling molecules such as nuclear hormone receptors, tyrosine and serine/threonine kinase. Regulators of cell death are critical for the cell signal sending networks^63^. Results of a study showed that the overexpression of the mitochondria heat shock protein 70 (mtHsp70) in protoplasts of transgenic rice affected the PCD^62^. HSP70 interacts with the members of the Bcl-2 family and prevents cell apoptosis^64^. HSP70 is usually required for polypeptide movement across the mitochondria inner membrane and further reactions of protein folding in the matrix^65^. The redox conditions of thiol-containing molecules (TRXs and GRXs) are important for cell performances such as synthesis, folding and structural regulation of proteins and transcription factors^62^.

These results indicate that an important part of the salt tolerance mechanism cannot be determined using GO analysis. To better understand the mechanisms of salt tolerance mediated by candidate genes, network analysis was utilized and sub-networks involving a large number of candidate genes and lowest edges were identified. The advantage of this method is that all molecular information available on quinoa as well as information obtained from other gene expression studies, can be utilized in an interactive network to extract more useful results and comperhensive understanding.

## Conclusion

In the present study, first, the genes responding to salinity stress in quinoa were identified and some were further analyzed for the first time. The assembled transcript was used to investigate differential expression and annotation of genes. We identified 3363 genes with differential expressions based on FDR < 0.001 and FC ≥ 2. The differential expression pattern for six of these genes was confirmed by qRT-PCR analysis and each demonstrated a similar level of up- or down- regulation. In the second part of the study, the reconstruction of the network of genes and the interaction of related proteins led to the identification of hub genes at 13.8 dsm^−1^ (HSPs family). These genes are expected to be essential in salt tolerance and it may be concluded that they can increase the tolerance threshold of quinoa to salt stress either individually or together. Among these genes, the effective role of WRKYs, bZIPs and MYBs may also be mentioned. Ontology analysis of the genes responding to salinity and hub genes revealed that protein processing in the endoplasmic reticulum is an important pathway involved in this stress. The protective effect of HSPs/chaperones may be attributed to the chaperone mechanism network in which many chaperones act in a coordinated manner. Under stress, many structural proteins are subjected to negative structural and functional changes. Therefore, refolding of the denatured proteins and maintenance of their function is critical for the survival of cells under stress conditions. These findings may be promising to update our knowledge about the role and changes in the genes involved in salt tolerance. This knowledge may be applied to the cultivation of halophytic plants such as quinoa using non-conventional water sources.

## Materials and methods

At all stages, the research complied with relevant institutional, national, and international guidelines and legislation.

### Plant material and salinity treatments

To investigate the transcriptome of quinoa under salinity stress, Titicaca genotype which is early-maturing and tolerant to adverse environmental conditions such as drought, cold and salinity^66,67^ was purchased from the Iranian National Salinity Research Center, Yazd, Iran. Salinity stress at 6.9 dSm^−1^ (1:1 sea water and double distilled water), 13.8 dSm^−1^ (sea water) and control (double distilled water) were applied with four biological replicates. The seawater originated from the Caspian Sea. Each pot contained loamy soil and a mixture of sand and humus (2:1). Salinity treatments were applied at the end of two leaf stage. The leaves were sampled at the end of four-leaf stage (30 days after emergence) 6 h, 1, 2, 3, 4, 5, 6 and 7 days after treatment. The samples were then immediately frozen in liquid nitrogen and kept at −80 °C.

### RNA extraction, cDNA library construction and sequencing

Total RNA was extracted using p-BIOZOL kit (Bioflax, Japan) from the samples and then treated with *DNase* I enzyme. Quantity and quality of the extracted RNA was confirmed using spectrophotometry at 260 nm and 1.5% agarose gel. Since parameters related to the enzymes and biochemical attributes (data not presented in the paper) were higher 4 days after treatment with 13.8 dSm^−1^, this treatment was selected along with the control to investigate the profile of total transcripts. This was done at BGI company (Shenzhen, China) using RNA-seq and NextFlex kit. Construction of cDNA library was done on 2500 Illumina Hi seq ™ 2500 (Illumina, USA) platform. Two cDNA libraries were constructed from the mRNA extracted from the control and 4 days after treatment (13.8 dSm^−1^) leaves of quinoa. Measurement with the Bioanalyzer instrument showed that all samples had RIN values of > 7.5 and therefore suitable for the construction of cDNA library and sequencing.

### Data analysis

Raw data underwent quality control and were edited using FastQC and Trimmomatic software. Reads with adapter sequences were omitted. Also, reads with low quality or unknown bases of > 5% were filtered to obtain high quality reads^68^. The sequence of *Chenopodium quinoa* transcript was downloaded from https://plants.ensembl.org/Chenopodium_quinoa/Info/Index. Then, the filtered reads along with the quinoa genome as the reference and Gene annotation were entered into STAR (v 2.7) software. High quality reads were mapped on the reference quinoa genome and transcriptomes were assembled. The role of genes were identified on Ensembled Plants (https://plants.ensembl.org/index.html) database. Function of novel genes was investigated using NCBI non-redundant (NR) database and BLASTp software. Identification of differential expression genes (DEG) in the samples was done using the R (v 4.1.2) software (https://www.rstudio.com/tags/website/). For this purpose, after the normalization of expression using edgeR package with trimmed mean of M-values normalization (TMM) method, Log_2_ value of Fold Change index was obtained for each gene. DEGs with FC values of ≥ 2 and with FDR of < 0.001 were considered as significant. Gene ontology (GO) was used to categorize the expressed functional DEGs. To categorize the genes according to their molecular role, cellular compartment and biological process, the list of GOs of DEGs were analyzed using AgriGO and g:profiler (http://biit.cs.ut.ee/gprofiler/) online software. Pathway enrichment analysis was done using the (https://string-db.org/cgi/input?sessionId=bOY6Uufuj0j2&input_page_active_form=multiple_identifiers). database. Important pathways were selected using Fisher’s exact test at and FDR of < 0.001.

### Confirmation of RNA-seq results using qRT-PCR

To confirm RNA-seq results, 6 DEGs were selected and primers were designed using Primer3 software (https://www.primer3plus.com/) based on the three prime untranslated (′3- UTR) regions. The names and sequences of primer used for real-time PCR amplification are listed in Table 5. Quantitative PCR was done using SYBR Green dye and SYBR BioPars kit (Gorgan University of Agricultural Sciences and Natural Resources, Iran) in IQ5 (Biorad, USA) real-time machine for three biological replicates. Produced cDNAs were then normalized using glyceraldehyde-3-phosphate dehydrogenase (GAPDH) household gene. Optimum conditions for qRT-PCR were done at 20 µl volume with three technical replicates for each sample. Data analysis was done using 2^−ΔΔCT^ using REST software^69^ method. Validation of the results of qRT-PCR and RNA-seq was then estimated using R software. Excel software was used to generate the related figures.

**Table 5:** Primer names and sequences used for real-time PCR amplification.

Primer name	Sequence	Tm	GC%	Product size (bp)
*CML39*	Forward 3'-TTGGTAGGTTGATGCAAGGC-5'	57.30	50	150
	Reverse 3'-GCTCACCAAGTCGAGTCAAC-5'	59.35	55	
*CBSX5*	Forward 3'-ATTCTTCCTCCGCGTCCTC-5'	58.83	57.89	127
	Reverse 3'-ATTGCCTCCGCCTTTCTGA-5'	56.67	52.63	
*TRX1*	Forward 3'-CGGAGGCATGGGAAGATCA-5'	58.83	57.89	121
	Reverse 3'-GCAAGCTCAGCCAGGAAAG-5'	58.83	57.89	
*GRXC9*	Forward 3'-TCCTTTGGTGTTTGTGGGTG-5'	57.30	50	101
	Reverse 3'-CGCCGGCTTGTTTGAGAATA-5'	57.30	50	
*SnRKγ1*	Forward 3'-TAGCCAGCAATGAGGACAGC-5'	59.35	55	136
	Reverse 3'-AGGGTGTCGGGTTCTTTGTG-5'	59.35	55	
*BAG6*	Forward 3'-TGGGAAGGAGCAGCAAGAA-5'	56.67	56.63	141
	Reverse 3'-GCAGGTTTCCCACACGAAG-5'	58.83	57.89	
*GAPDH*	Forward 3'-GGTTACAGTCATTCAGACACCATCA-5'	62.40	44	122
	Reverse 3'-AACAAAGGGAGCCAAGCAGTT-5'	62.37	59.09	

### Network reconstruction

To draw the gene network and find hub genes among DEGs with FC > 2, orthologue genes of quinoa in *Arabidopsis thaliana* from g: Profiler were used. Then, its protein–protein interaction network was generated using the STRING database and Confidence 0.4. This network was then reconstructed in Cytoscape software. To generate the protein interaction network and identify the genes influencing salinity tolerance in this network, Cytohubba plugin was used in Cytoscape software^27^. To identify hub genes (10 nodes with the highest interaction) in the network, four Cyto-Hubba calculation algorithms (MCC, DMNC, MNC and Degree) were used.

Information from gene ontology and pathways of DEGs with FC > 2 and hub genes (identified by the four Cytohubba algorithms) were extracted using Agri GO software and STRING database. The, Cytocluster plugin was then used for better clustering of the subnetworks obtained from Cytohubba plugin (IPCA algorithm)^27^. This algorithm categorizes the clusters regarding the protein complex and is more operational than the other algorithms in this App^27^. GO pathway was extracted from each cluster using the STRING database.

## Data Availability

The datasets generated and/or analysed during the current study are available in the SRA NCBI repository, http://www.ncbi.nlm.nih.gov/bioproject/915188. Submission ID: SUB12432421. BioProject ID: PRJNA915188. SAMN32379043 SH-CTRL-R2 SH-CTRL-R2 *Chenopodium quinoa* 63459 Titicaca. SAMN32379044 SH-4d-R2 SH-4d-R2 *Chenopodium quinoa* 63459 Titicaca. https://www.ncbi.nlm.nih.gov/biosample/32379043. https://www.ncbi.nlm.nih.gov/biosample/32379044.
